# Difficulties capturing co-occurring traumatic brain injury among people with traumatic spinal cord injury: a population-based study

**DOI:** 10.1038/s41393-022-00851-5

**Published:** 2022-09-16

**Authors:** Deborah L. Snell, Julianne Johns, Jennifer A. Dunn, Joanne L. Nunnerley, Balraj Singhal

**Affiliations:** 1 Department of Orthopaedic Surgery and Musculoskeletal Medicine, University of Otago, PO Box 4345, Christchurch, 8140 New Zealand; 2 Burwood Academy, Private Bag 4708, Christchurch, 8140 New Zealand; 3 Burwood Spinal Unit, Te Whetu Ora Waitaha Canterbury, Christchurch, New Zealand

**Keywords:** Health services, Spinal cord diseases, Brain injuries

## Abstract

**Study design:**

This is a population-based prospective cohort study.

**Objectives:**

Traumatic brain injury (TBI) is common among people with traumatic spinal cord injury (TSCI), but rates vary across studies associated with variable approaches to diagnosis. We aimed to determine if a published diagnostic algorithm could be consistently applied to capture co-occurring TBI among persons sustaining TSCI.

**Setting:**

One of two spinal centres in New Zealand (NZ), the Burwood Spinal Unit (BSU) captures approximately 45% of NZ TSCI admissions.

**Methods:**

Adults (age 16+) with TSCI admitted to the BSU between 1 January 2021 and 31 August 2021 (*n* = 51) were included. Clinical notes were audited prospectively to identify co-occurring TBI.

**Results:**

We identified co-occurring TBI in 39% of TSCI cases with a small number of additional suspected TBI cases where TBI could not be confidently ruled in or out. Including all TBI cases, suspected or otherwise resulted in up to 55% of the sample having sustained co-occurring TBI. There were difficulties applying the published algorithm, associated with inconsistent documentation of TBI indicators from acute to rehabilitation contexts.

**Conclusions:**

In this study, the feasibility of a TBI diagnostic algorithm for the TSCI population was low. Alternative approaches to screening for TBI among people sustaining TSCI are needed. Greater consistency in documenting TBI across the continuum of care will ensure TBI if present, is included in treatment planning.

## Introduction

Traumatic brain injury (TBI) is a common co-occurring condition associated with traumatic spinal cord injury (TSCI), with potentially significant implications for rehabilitation [1]. Co-occurring TSCI and TBI have been associated with increased length of rehabilitation [2], added difficulties with psychosocial functioning and adjustment [3], and neurocognitive impairment [4]. Missed TBI symptoms can be perceived as non-compliance, inability to learn or benefit from rehabilitation, maladaptive reactions to TSCI, or poor motivation [1, 5]. Co-occurring TSCI and severe TBI have been associated with worse motor outcomes, longer acute rehabilitation length of stay, and lower gains from admission to discharge [4].

Even when the TBI falls in the mild range, there can be implications for rehabilitation progress and functional status at discharge. Symptoms that are highly prevalent in the early months even after mild TBI, such as fatigue, dizziness, cognitive difficulties and emotional regulation disturbances [6, 7], may delay the person’s ability to tolerate and benefit from intensive early inpatient rehabilitation [8]. In addition, persisting unrecognised TBI impacts beyond inpatient rehabilitation could be evident in difficulties with adjustment and higher level executive demands of returning to life in the community with an SCI as well as returning to employment [9]. When a TBI is missed, there is greater risk of misattributing such difficulties to other factors such as reduced motivation, poor adjustment to SCI and depression [5].

Although there are only a handful of studies, rates of co-occurring TSCI and TBI vary considerably with estimates ranging from 25–70% of cases [1, 8]. Some studies have found that TBI is missed in up to half of persons with TSCI [10]. As in the non-SCI population, the majority of co-occurring TBI’s fall in the mild range of severity. Case ascertainment difficulties and variability in methods for identifying co-occurring mild TBI in particular, may contribute to variable estimates of TBI incidence [5]. Challenges detecting mild TBIs include subtle or absent neurological signs and symptoms on admission to acute services in a context of more pressing life threatening injuries [11]. Available diagnostic tests lack sensitivity for mild TBI, such as commuted tomography (CT) [6]. Other possible TBI signs such as eye co-ordination symptoms can be difficult to reliably detect acutely although developing technologies show promise [12]. Some work has also suggested self-reporting TBI indicators may be unreliable, so self-report TBI assessment tools may be unhelpful for capturing co-occurring TBI [1].

Agreed upon algorithms for capturing co-occurring TBI that are based on multiple indicators are needed [1, 2]. Macchiocchi, Seel, Thompson, Byams and Bowman [2] developed an algorithm prioritising estimated duration of post traumatic amnesia (PTA) before tracking variations associated with neuroimaging outcomes and the Glasgow Coma Scale (GCS) score. Using such an algorithm may improve consistency and reliability of capturing co-occurring TBI diagnosis and improve accuracy of incidence estimation.

In New Zealand (NZ) rates of co-occurring TSCI and TBI are largely unknown. With the establishment of the NZ Spinal Cord Injury Register in 2017 (NZSCIR: www.nzspinaltrust.org.nz/nzscir), there is an opportunity to address this gap. Our preliminary work reviewing 18 months of retrospective NZSCIR data (*n* = 298) highlighted missing TBI data and reliance on single problematic injury indicators such as the GCS score. Resulting TBI estimates varied and ranged between 44.8% (field) to 49.8% (facility) of TSCI cases but severity indicators were incomplete and difficult to verify [13].

The objective of this study was to audit prospective cases admitted to a spinal rehabilitation centre in NZ to evaluate rates of co-occurring TSCI and TBI, using the algorithm developed by Macchiocchi et al. [2]. We aimed to determine if such an algorithm could be consistently applied to capture TBI.

## Methods

### Design and setting

This is an audit of prospective admissions to one of the two spinal rehabilitation centres in NZ, the Burwood Spinal Unit (BSU). The BSU captures approximately 45% of all TSCI admissions in NZ, with a catchment area extending across both the North and South Islands of NZ. The study received ethical approval from the University of Otago Human Ethics Committee (ref HD 20/046).

### Participants

All adults (aged 16+) sustaining TSCI between 1 January 2021 and 31 August 2021 who were admitted to the BSU were eligible. Cases of paediatric SCI (<age 16) were not included. This is because in NZ children with SCI are often admitted to a designated children’s hospital in another centre. Cases sustaining SCI from non-traumatic mechanisms were also excluded.

### Procedure for TBI case ascertainment

Determination of TBI followed the algorithm established by Macchiocchi and colleagues [2]. First, we looked for evidence of PTA, then looked for other TBI indicators such as GCS scores and neuroradiological findings. Severity classifications for TBI severity were based on widely accepted diagnostic criteria [14, 15]. The criteria used for TBI capture are shown in Table 1.

**Table 1:** Classification criteria for traumatic brain injury presence and severity^1^.

TBI Classification	PTA duration	GCS score	Intracranial lesion evident
No TBI	None evident	15	No
Mild TBI	<24 hours	13–15	No
Mild TBI with neuroimaging evidence of structural intracranial lesion	<24 hours	13–15	Yes
Moderate TBI	1–6 days	9–12	Yes or No
Severe TBI	≥7 days	3–8	Yes or No

^1^Criteria set from Menon et al. [14]. and American Congress of Rehabilitation Medicine Special Interest Group [15].

*TBI* traumatic brain injury, *PTA* post traumatic amnesia, *GCS* Glasgow Coma Scale score.

Suspected TBI cases were those where there was a biomechanically plausible TBI mechanism but where it was unclear whether documented signs and clinical findings of TBI present were accounted for by confounding factors. “Suspected” means that a TBI was possible or probable but clinical signs were difficult to ascertain or there was insufficient information or confounding factors that prevented a higher level of diagnostic certainty.

### Data collection and procedure

Adults with TSCI admitted to the BSU were screened for co-occurring TBI. Information extracted from clinical records included demographic variables (age, sex, ethnicity) and clinical variables. Clinical variables included mechanism and context of TSCI, International Standards for Neurological Classification of Spinal Cord Impairment (ISNCSCI) classification [16], and TBI indicators where available. Indicators of TBI included duration of PTA, GCS scores, references to loss of consciousness or altered mental state, and neuroimaging reports.

Evidence of co-occurring TBI was sought in prehospital electronic and paper documents (including emergency medical services, ambulance, and transfer records), radiology reports, first hospital acute service and intensive care admission and transfer notes, and inpatient rehabilitation admission and clinical notes. This included references to presence and length of PTA, loss of consciousness, lowest GCS score within 24 h after injury or post-extubation (if intubated), neuroradiology results, clinician reports of altered mental status, and any diagnoses of TBI. Cases were identified where risk factors for co-occurring TBI were evident. These risk factors included (i) mechanism of injury, such as fall and motor vehicle accident, and (ii) cases of cervical level TSCI, because evidence notes associations between such factors and increased risk for TBI in the TSCI population [1]. References in clinical notes to changes in cognition, emotional regulation, personality, and other post-concussion type symptoms such as dizziness may be indicators of TBI and were also examined.

### Data analyses

Data were analysed using descriptive statistics such as means, standard deviations for continuous variables, and frequencies (n (%)) for categorical variables. No specific approaches were taken to manage missing data in line with the objectives of the study. Rather where there were missing data points these are reported.

## Results

### Description of study sample

Demographic and clinical information for the sample is shown in Table 2. A total of 51 persons with TSCI were included in the study. The mean age of the sample was 49 years and there were more men than women. The most common injury mechanisms were transport-related, sport-related and falls. Table 2 shows those with cervical level TSCI’s were over-represented among those with a TBI.

**Table 2:** Demographic and clinical characteristics of the study sample (*n* = 51).

	TBI (yes)^1^ (*n* = 28)	TBI (No) (*n* = 23)
**Demographic characteristics**		
Age [(years), mean (SD), range]	49.8 (20.1), 16–91	48.2 (20.4), 16–83
Age in groups [*n* (%)]^*a*^		
-16–29 years	6 (21.4)	5 (21.7)
-30–44 years	6 (21.4)	5 (21.7)
-45–59 years	5 (17.9)	6 (26.1)
-60–74 years	9 (32.1)	4 (17.4)
-75+ years	2 (7.1)	3 (13.0)
Sex [male, *n* (%)]	23 (82.1)	14 (60.9)
Ethnicity [*n* (%)]^*b*^		
-New Zealand European	17 (60.7)	13 (56.5)
-New Zealand Māori	4 (14.3)	3 (13.0)
-Other	2 (7.1)	5 (21.7)
**Clinical Characteristics**		
Level and severity of TSCI [n (%)]		
-C1- 8 AIS A, B, C	18 (64.3)	6 (26.1)
-T1–S3 AIS A, B, C	8 (28.6)	6 (26.1)
-AIS D at any level	2 (7.1)	11 (47.8)
Mechanism of tSCI [n (%)]		
-Transport-related	11 (39.3)	6 (26.1)
-Sport-related	8 (28.6)	7 (30.4)
-Fall	7 (25.0)	7 (30.4)
-Assault	1 (3.6)	0 (0.0)
-Other	1 (3.6)	3 (13.0)

^*a*^Groups defined following Biering-Sørensen et al. [21] ^*b*^Numbers do not total 51 because of missing data.

^1^TBI (yes) includes confirmed and suspected cases.

*TSCI* traumatic spinal cord injury, *AIS* American Spinal Injury Association Impairment Scale, *C* cervical, *T* thoracic, *S* sacral, *GCS* Glasgow Coma Scale, *TBI* traumatic brain injury.

### TBI ascertainment

Table 3 shows the information available for capturing co-occurring TBI. Notably references to PTA were evident in just under a third of cases and in most of these, PTA was either documented as absent or duration of amnesia could not be estimated from the records. In contrast a recorded GCS score within the relevant timeframe (within 24 h after injury or post-extubation) was available in two-thirds of the cases reviewed. Given the wide catchment area for the BSU, some admissions in the period of the audit included persons living in other regions of NZ. Paramedic records for these persons were not available to us. Other variables that might be indicative of co-occurring TBI such as references to disorientation, altered mental state, or loss of consciousness were not recorded in sufficient detail to usefully ascertain TBI.

**Table 3:** Traumatic brain injury indicators (*n* = 51).

Variable	*N* (%) or Mean (SD)
PTA documented [(yes) *n* (%)]	16 (31.4)
PTA duration [*n* (%)]	
-None	11 (21.5)
-< 24 hours	2 (3.9)
->24 h <7 days	1 (2.0)
->7 days	2 (3.9)
-Unable to estimate or missing	35 (68.6)
GCS score documented [(yes) *n* (%)]	34 (66.7)
GCS score [mean (SD), range]	13.4 (3.1), 3-15
GCS score [*n* (%)]	
-GCS score 13–15	27 (52.9)
-GCS score 9–2	5 (9.8)
-GCS score 3–8	2 (3.9)
LOC documented or suggested [(yes) *n* (%)]	21 (42.0)
Disorientation documented [(yes) *n* (%)]	5 (9.8)
Neuroimaging evidence of structural intracranial injury [(yes) *n* (%)]	9 (17.6)
Co-occurring TBI identified [*n* (%)]	
-Yes	20 (39.2)
-No	23 (45.1)
-Suspected but insufficient information or confounding factors present	8 (15.7)
TBI Severity Estimate [*n* (%)]^1^	
-No TBI	23 (45.1)
-Suspected mild TBI	8 (15.7)
-Mild TBI	10 (19.6)
-Mild TBI with neuroimaging evidence of structural intracranial injury	5 (9.8)
-Moderate^*a*^	2 (3.9)
-Severe^*b*^	3 (5.9)

^*a*^*n* = 2 cases with reduced GCS were confounded by substance intoxication and *n* = 1 invalid GCS secondary to impaired motor response during testing were downgraded to mild range. ^*b*^Includes *n* = 1 with no GCS but diagnosis of severe TBI in notes.

*PTA* post traumatic amnesia, *GCS* Glasgow Coma Scale, *LOC* loss of consciousness, *TBI* traumatic brain injury.

^1^TBI criteria from Menon et al. [14]. and American Congress of Rehabilitation Medicine Special Interest Group [15].

With the data available to the research team a confident determination of co-occurring TBI was able to be made in 39% of cases. Table 3 shows there was a proportion of cases (*n* = 8) where TBI was suspected because the recorded mechanism and/or indicators were suggestive of TBI but confounding factors such as substance intoxication were evident. This means up to 55% of TSCI admissions during the period of the audit may have sustained a co-occurring TBI. Of the 28 TBI cases (including suspected cases), *n* = 23 were designated as mild TBIs. When information was found that enabled capture of co-occurring TBI this was not always able to be tracked consistently across the patient journey from first hospital admission to the rehabilitation facility (Table 4).

**Table 4:** Number of cases where information to identify TBI was found (*n* = 28).

Variable	*N* (%)
Paramedic notes^*a*^	6 (21.4)
Intensive Care handover notes	8 (28.6)
First Hospital admission/transfer notes	17 (60.7)
BSU inpatient admission notes	17 (60.7)
BSU inpatient clinical notes and/or treatment plans	11 (39.3)

^*a*^Paramedic notes were unavailable for out of region injuries; *TBI* traumatic brain injury, *BSU* Burwood Spinal Unit.

## Discussion

In this study we sought to identify co-occurring TBI among persons with TSCI admitted to a spinal rehabilitation centre in NZ. Our aim was to evaluate the feasibility of a published algorithm for capturing co-occurring TBI. We were unable to consistently apply the algorithm because of variable documentation of TBI indicators. However by careful notes review we were able to identify co-occurring TBI in 39% of TSCI cases with a small number of additional suspected TBI cases where TBI could not be confidently ruled in or out based on the information available. Including all cases of TBI, suspected or otherwise resulted in up to 55% of the sample having sustained co-occurring TBI. This seems consistent with the small number of international studies reporting co-occurring TBI rates [1, 8]. In addition, it has been reported that among persons with TSCI, 30–34% also sustain a mild TBI, 11–16% sustain a moderate TBI and 6–10% sustain severe TBI [1]. While broadly echoing the patterns in our sample, some variability associated with the way we incorporated neuroimaging findings may have resulted in a higher proportion of mild TBI and lower proportion of moderate TBI relative to other published studies. Acute neuroimaging such as CT is not routinely recommended after mild head trauma with exceptions according to widely used head CT rules [6]. While the role of magnetic resonance imaging for evaluating the injured spine following TSCI is reasonably well established [17], this is less established and lacks sensitivity for acute management of TBI, especially for cases in the mild range of severity [18]. In our sample, neuroradiology findings, positive or negative, were available for only a small number of cases, perhaps reflecting these issues.

Applying the algorithm published by Macchiocchi et al [2]. was not feasible because of the inconsistent recording of references to PTA in particular, as well as other TBI signs and indicators. When references were made to PTA or amnesia it was difficult to confidently estimate duration. The GCS score was not able to be found in a third of cases and references to loss of or altered consciousness were patchy at best. This is not unique to the study context and reinforces issues with inconsistent screening for TBI noted by others [19]. These are issues that will impact clinical uptake of such an algorithm by clinicians.

Turning to the mild TBI literature and diagnostic guidelines may be helpful and especially relevant given most issues with consistency of identifying co-occurring TBI are associated with injuries in the mild range [1, 5]. TBI in the moderate and severe range may be more obvious. In a synthesis of mild TBI practice guidelines [6], a three-step approach to TBI diagnosis was suggested. First and critically, clinicians should establish a plausible mechanism, where sufficient biomechanical energy is evident to disrupt brain function. While commonly reported but not always consistently found across studies [8], cervical level injuries should flag a co-occurring TBI diagnosis. Other mechanistic risk factors for co-occurring TBI such as transport injuries and falls have been identified reasonably consistently in other studies [5]. These are potentially useful starting points for routine screening for TBI among persons with TSCI, and especially those with cervical spinal cord injury.

The second step is to query signs and symptoms of altered mental status and the third step underscores the importance of considering confounding factors, such as substance intoxication, acute psychological stress, and treatments administered early after injury. At steps two and three, TBI indicators such as the GCS score, PTA if available, other evidence of altered mental status as available should be considered in the context of confounds. Such a three step approach underscores the importance of looking for multiple converging sources of information to capture TBI co-occurring with TSCI, notwithstanding documentation problems as described.

One other issue our study highlighted was the inconsistency with which TBI indicators travelled with the individual as they progressed from the injury site to the first hospital and then the rehabilitation centre. This has been noted as a significant area of concern by others and emphasises the need for more standardised screening processes to ensure co-occurring TBI is consistently communicated across the continuum of care [19].

Our study underscores the need for a consistent approach to capturing co-occurring TBI. The three step approach outlined above incorporating multiple sources of information to reliably capture and then consistently communicate co-occurring TBI may have promise [19]. The larger issue however is how such approaches can be incorporated into clinical practice. There is a body of evidence describing barriers and difficulties with uptake of evidence-based guidelines in clinical practice [20], and this is a focus of our ongoing work.

### Limitations

This is a small sample from a single centre albeit with a wide nationally representative catchment area. Further, while the number of suspected TBI cases was relatively small, including these in total figures for TBI can inflate estimates because of possible false positives [8]. The figures we report should be considered with this caveat in mind.

Inability to access paramedic records resulted in missing information because a large proportion of cases sustained injuries in other regions and paramedic records did not travel with the individual to the rehabilitation facility. This is not likely to be a significant issue if initial GCS information and references to altered mental state are captured reliably elsewhere.

## Conclusions

This study focused on improving consistency of capturing co-occurring TBI among persons with TSCI in NZ. We highlighted the difficulties associated with inconsistent documentation of TBI indicators such as PTA and GCS scores from acute to rehabilitation contexts. These documentation issues meant applying an algorithm relying on such indicators was not feasible. Alternative approaches to screening for TBI among people sustaining TSCI are needed. Greater consistency in documenting TBI indicators across the continuum of care will ensure TBI if present, is included in treatment planning.
